# Discovery Strategies of Bioactive Compounds Synthesized by Nonribosomal Peptide Synthetases and Type-I Polyketide Synthases Derived from Marine Microbiomes

**DOI:** 10.3390/md14040080

**Published:** 2016-04-16

**Authors:** Grigoris D. Amoutzias, Anargyros Chaliotis, Dimitris Mossialos

**Affiliations:** Department of Biochemistry & Biotechnology, University of Thessaly, Larissa 41221, Greece; amoutzias@bio.uth.gr (G.D.A.); anargyros.chaliotis@gmail.com (A.C.)

**Keywords:** bioactive compounds, bioprospecting, marine microbiomes, evolution, genome mining, metagenomics, nonribosomal peptide synthetase, polyketide synthase

## Abstract

Considering that 70% of our planet’s surface is covered by oceans, it is likely that undiscovered biodiversity is still enormous. A large portion of marine biodiversity consists of microbiomes. They are very attractive targets of bioprospecting because they are able to produce a vast repertoire of secondary metabolites in order to adapt in diverse environments. In many cases secondary metabolites of pharmaceutical and biotechnological interest such as nonribosomal peptides (NRPs) and polyketides (PKs) are synthesized by multimodular enzymes named nonribosomal peptide synthetases (NRPSes) and type-I polyketide synthases (PKSes-I), respectively. Novel findings regarding the mechanisms underlying NRPS and PKS evolution demonstrate how microorganisms could leverage their metabolic potential. Moreover, these findings could facilitate synthetic biology approaches leading to novel bioactive compounds. Ongoing advances in bioinformatics and next-generation sequencing (NGS) technologies are driving the discovery of NRPs and PKs derived from marine microbiomes mainly through two strategies: genome-mining and metagenomics. Microbial genomes are now sequenced at an unprecedented rate and this vast quantity of biological information can be analyzed through genome mining in order to identify gene clusters encoding NRPSes and PKSes of interest. On the other hand, metagenomics is a fast-growing research field which directly studies microbial genomes and their products present in marine environments using culture-independent approaches. The aim of this review is to examine recent developments regarding discovery strategies of bioactive compounds synthesized by NRPS and type-I PKS derived from marine microbiomes and to highlight the vast diversity of NRPSes and PKSes present in marine environments by giving examples of recently discovered bioactive compounds.

## 1. Introduction

**Figure 1:**
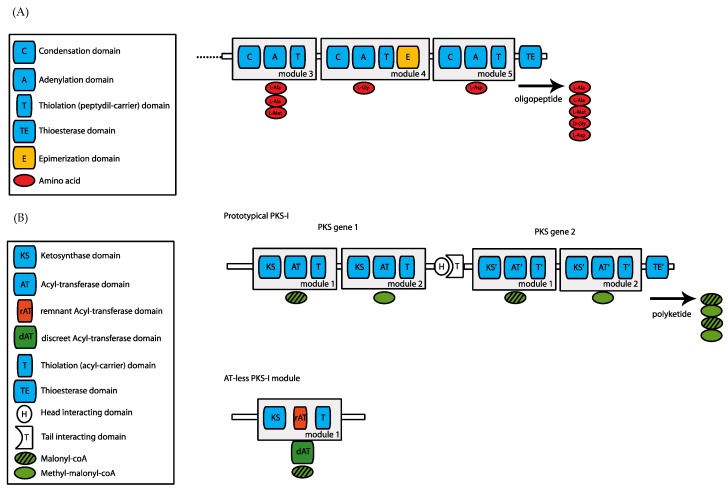
Module organization of nonribosomal peptide synthetases and type-I polyketide synthases. (**A**) A typical module of NRPSes consists of three main domains: C, A and T. The condensation domain (C) is responsible for the formation of the C–N bond between the elongated chain and the activated amino acid. The adenylation (A) domain activates its related amino acid and catalyzes the transfer of the activated substrate to thiolation (peptidyl-carrier) (T) domain of the same module. The epimerization domain (E) is an auxiliary domain that changes an l-amino acid into a d-amino acid. Each module is responsible for the incorporation of one monomer in the elongated oligopeptide. TE domain releases the final peptide product from the enzyme through cyclization or hydrolysis; (**B**) A prototypical module of type-I PKSes consists of the following domains: KS, AT and T. The AT domains are responsible for the incorporation of malonyl or methylmalonyl-CoA monomers, while the KS domains form a C–C bond. Acyl carrier (T) domains are equivalent to PCP (T) domains of NRPS. Each module is responsible for the incorporation of one monomer in the elongated polyketide. TE domain releases the final polyketide product from the assembly line. Type-I PKS proteins may interact in a head to tail fashion, thus forming a megasynthase. AT-less PKSes-I are characterized by the absence of integrated AT domains within each extension module. Instead, a free-standing AT domain named discreet AT acts in-*trans* through binding to the remnant AT domain within the module.

Oceans were the cradle of life and they still host an enormous biodiversity which is rather under-explored. The exponential advances in new technologies and engineering have opened up the marine ecosystems to scientific endeavors aiming to discover valuable novel metabolic compounds. Marine natural product discovery was initially focused on the easily accessible macroorganisms such as algae, corals, molluscs and sponges. However, efforts have gradually focused on microbes such as bacteria and fungi, which constitute a large portion of marine biodiversity. Due to metabolic versatility, there is a great potential harbored within microorganisms to produce bioactive compounds for a wide range of applications, particularly in medicine. However, traditional culture-based approaches fail to identify the majority of bioactive compounds produced by microorganisms. It is estimated that roughly 1% of bacteria are cultured *in vitro*, whereas 31 of the 61 known bacterial phyla lack cultivable representatives. Marine microorganisms are free-living or associated with other organisms, particularly invertebrates. They produce a large repertoire of pharmaceutically and biotechnologically useful metabolites including antimicrobial, anticancer, anti-inflammatory, antioxidant compounds, antifouling agents and nutraceuticals [1,2,3,4]. In many cases, secondary metabolites like nonribosomal peptides (NRPs) and polyketides (PKs) act as antibiotics, immunosuppressants, antitumor agents, toxins and siderophores. They are synthesized by multimodular enzymes named nonribosomal peptide synthetases (NRPS) and type-I polyketide synthases (PKS-I), respectively [5,6,7,8,9]. Studies on NRPS and type-I PKS demonstrated an analogy regarding their architecture and function (Figure 1). NRPSes are built of catalytic units called modules [10]. Each module is 1,000−1,100 amino acids long and, according to the colinearity rule, the number and the order of the modules defines the number and the order of amino acids in NRPs [7,8]. Enzyme modules contain three catalytic domains: In NRPS the adenylation (A) domain is responsible for recognition and activation of an amino acid followed by transfer of the activated substrate to the peptidyl-carrier (thiolation) domain of the same module. Finally, the condensation (C) domain of the downstream module forms the C-N bond between the elongated chain and the activated amino acid [10,11,12]. In some cases, a module may incorporate additional auxiliary domains, such as the epimerization (E) domain which changes an l-amino acid into a d-amino acid as well as the dual/epimerization domains (E/C) responsible for both epimerization and condensation [11]). Cyclization (Cy) domains have also been detected in several NRPS gene clusters. These domains can replace C-domains responsible for the incorporation of cysteine, serine or threonine. The oxidation (Ox) domain is usually located either downstream of the PCP-domain or in the C-terminus of the A-domain and catalyzes the formation of an aromatic thiazol through oxidation of a thiazoline ring [7,13]. Thioesterase domains (TE) are usually located in the final NRPS module [7,14]. TE domains release the final peptide product from the enzyme through cyclization or hydrolysis [10,15,16].

In a structural and functional analogy to NRPS, the PKS-I modules contain three catalytic domains: the acyl-transferase (AT), ketosynthase (KS) and acyl-carrier (thiolation) domains. The AT domains incorporate malonyl or methylmalonyl-CoA, while KS domains form a C–C bond. The ACP domain is equivalent to the peptidyl-carrier (PCP) domain of NRPS. The ketoreduction (KR) and dehydration (DH) domains are auxiliary domains of PKS [16]. Also, enoylreductase (ER) domains have been observed in PKS modules and they perform further reduction of the C-C double bonds [7,8]. Whereas all of the aforementioned PKS domains are found in prototype modular PKSes, there is an emerging family of *trans*-AT or AT-less PKSes. The defining feature of such PKSes is the absence of integrated *cis*-acting AT domains within each PKS extension module. This essential substrate loading activity is instead provided in-*trans* through the action of free-standing *trans*-acting ATs encoded within the synthase gene cluster [17,18,19].

A gene cluster with both NRPS and PKS encoding genes leads to the formation of hybrid NRPS/PKS-derived products. Hybrid systems interact in a head to tail fashion and their order modifies the structure of the bioactive compound. Bioactive compounds synthesized by hybrid NRPS-PKS systems can be divided into two main classes. The first one includes natural products synthesized individually by NRPS and PKS and eventually coupled into a hybrid final product. In the second class the NRPS and PKS enzymes are functionally connected thus leading directly to a hybrid peptide-polyketide metabolite [7,8,20].

Recent studies have demonstrated a possible link between ribosomal and nonribosomal synthesis of peptides. In normal ribosomal biosynthesis, aminoacyl-tRNA synthetases catalyze aminoacylation of tRNAs with a specific amino acid. However, in 2010, Mocibob *et al.* suggested that a group of atypical seryl-tRNA synthetase (SerRS)-like proteins found in diverse bacterial genomes could be involved in nonribosomal peptide synthesis [21]. This hypothesis is further supported by the functional homology between atypical SerRS homologs and adenylation domains in NRPS. Both types of enzymes catalyze a two-step reaction that includes the activation of an amino acid as well as the specific aminoacylation of eligible carrier proteins [21].

Additional evidence supporting the connection of typical ribosomal and nonribosomal peptide synthesis is offered from transferase PacB which participates in pacidamycin biosynthesis. PacB is a tRNA-dependent aminoacyltransferase that catalyzes the incorporation of alanine, derived from an alanyl-tRNA, to the N-terminal residue of a tetrapeptide intermediate. This tetrapeptide intermediate is assembled through a NRPS system and the final yielded product is a pentapeptidyl nucleoside antibiotic [22]. The above examples suggest that nonribosomal peptide synthesis is not completely unrelated to ribosomal protein synthesis, thus indicating an ancestral evolutionary link between these biosynthetic pathways.

The aim of this review is to examine recent developments regarding discovery strategies of bioactive compounds synthesized by NRPS and PKS-I derived from marine microbiomes and to highlight the vast diversity of NRPSes and PKSes present in marine environments by giving examples of recently discovered bioactive compounds.

## 2. Evolution of Nonribosomal Peptide Synthetases (NRPSes) and Type-I Polyketide Synthases (PKSes)

Recently, a systematic analysis of genomic data has shown that NRPS and type-I PKS biosynthetic pathways are widely distributed in bacteria and found sporadically in Archaea and Eukarya (fungi and metazoa) [23]. Intriguingly, NRPs and PKs are secondary metabolites of relatively small size, synthesized by the largest known enzymes found to be present in microbial genomes. This paradox raises a reasonable question: Why have microorganisms throughout evolution relied on such biosynthetic pathways of genes with modular architecture and extraordinary size that are so energetically costly? [8,24]. In the case of NRPSes a typical module of three domains that spans roughly 1,100 amino acids is responsible for the incorporation of one monomer in the elongated chain. Microorganisms synthesize huge enzymes (megasynthases) which means significant metabolic expenditure at the transcription and translation level in order to produce NRPs still capable of synthesizing peptides of comparable size through normal protein synthesis. A prominent example is bacteriocin synthesis [25]. Within the framework of this review, we performed a rudimentary bioinformatics analysis of 2,771 prokaryotic genomes, (explained in detail is section 4—Genome Mining). Our analysis revealed that the largest NRPS megasynthase is found in *Photorhabdus luminescens* (subsp. *laumondii TTO1*) with a size of 16,367 aa and 14 modules. Correspondingly, the largest PKS-I is found in *Mycobacterium liflandii* 128FXT with a size of 17,019 aa and nine modules.

The reason why microorganisms use such energetically inexpedient enzymes for the production of secondary metabolites is still elusive. A reasonable explanation could be that microorganisms take advantage of domain reshuffling in order to rapidly produce a vast repertoire of secondary metabolites or that secondary metabolites produced by NRPSes contain unusual amino acids that might not be easily incorporated into the final product through normal protein synthesis. After all, microbes that produce NRPs should have a protective or adaptive advantage in specific niches [8,26].

It has been demonstrated by comprehensive studies that NRPSes evolve by gene duplications, which can be intragenic or intergenic, in combination with domain insertions/deletions/reshuffling. Recombination events, horizontal gene transfer (HGT) or point mutations may also contribute to metabolite diversification [8,26,27,28]. Further diversity of NRPSes and PKSes is produced by the module-skipping mechanism, thus leading to metabolites with revised activities. Myxochromides are well-established lipopeptides produced from the Myxobacteria genus. A hybrid NRPS-PKS machinery forms the hexapeptide myxochromide A, while a structurally similar assembly line produces the pentapeptide myxochromide S [27]. It has been demonstrated that the fourth amino acid (l-proline) of myxochromide A does not exist in the myxochromide S product, indicating that one module is skipped during the biosynthesis process of myxochromide S. The proposed mechanism suggests that the biosynthetic intermediate is transferred to the peptidyl-carrier (PCP) domain of module 4 and the elongation process continues through the incorporation of alanine from the PCP domain of module 5 [7,29]. A typical example of module skipping in polyketides has been illustrated in biosynthetic studies of leinamycin (LNM), an antitumor agent. The LNM gene cluster encodes a hybrid NRPS-PKS system which consists of two NRPS and five PKS modules. However, PKS module-6 contains two acyl-carrier (ACP) domains (ACP_6-1_ and ACP_6-2_). Through the skipping mechanism, both ACP domains can replace each other during LNM biosynthesis, despite the fact that ACP_6-2_ seems to be preferred due to its higher loading efficiency [30]

Evolutionary mechanisms driving diversification of type-I PKSes in bacteria are gene duplications and losses, recombination events and horizontal gene transfers (HGT) [8,24,28]. HGT is a common phenomenon between bacteria and fungi as well as among bacteria and fungi leading to acquisition of novel NRPS or PKS-I gene clusters [8,24]. In a recent study, Slot *et al*. observed an unusual HGT phenomenon in *Aspergillus nidulans*, a filamentous fungus, which synthesizes polyketide-derived secondary metabolites including sterigmatocystin [31]. Sterigmatocystin gene cluster contains 23 genes that form the complete biosynthetic pathway. Evidence was shown that the whole gene cluster was transferred horizontally from *Aspergillus nidulans* to *Podospora anserina.* The gene cluster was still expressed, indicating that transfer of whole gene clusters between different taxonomic classes could lead in heterologous production of secondary metabolites [31].

Phylogenetic analysis of type-I PKSes based on AT domains indicated that PKS-I genes in bacteria were diversified in two distinct evolutionary clades. The first clade includes all AT domains which activate malonyl-CoA, including some domains without any predicted substrates, whereas the second clade includes AT domains activating methyl-malonyl-CoA or rare substrates. Gene duplications followed by subfunctionalization contributed further to the evolution of AT domains in the second clade [7,24]. Another phylogenetic analysis based on highly conserved keto-acyl synthase (KS) domains demonstrating the presence of two distinct clades containing exclusively *cis*- or *trans*-AT PKSes. The tight clustering of *trans*-AT PKSes regardless of the bacterial phylum indicates that these enzymes have a single evolutionary origin. Lack of tight phylogeny between *trans*-AT PKSes and their host organisms may indicate a higher rate of HGT and/or a higher tendency of these proteins to be expressed and folded correctly in new host strains after such transfers, compared with proteins of the *cis*-AT type [32].

Two recently published studies examined in detail the evolutionary processes of NRPS and PKS gene clusters present in marine bacteria [33,34]. In the first study, a phylum-wide investigation of genetic diversification of the NRPS and PKS biosynthetic pathways was carried out using genomic data from 126 cyanobacterial genomes. In total, 452 NRPS, PKS and hybrid NRPS/PKS gene clusters were identified from 89 cyanobacterial genomes, forming 286 highly diversified cluster families of pathways. Interestingly, only 20% of the gene clusters could be assigned to the described biosynthetic pathway of a natural product belonging to a known group of chemical compounds indicating that Cyanobacteria is a prolific source of new chemical scaffolds [30]. Roughly 4% of these gene clusters were located on plasmids, probably due to HGT. Another 26% of gene clusters were surrounded by or contained genes encoding mobile elements such as transposases, phages and integrases, potentially involved in HGT. However, the highly degraded state of mobile elements in the surrounding of gene clusters suggested that many of the HGT events were ancient. Nevertheless, HGT might not be the main driving force acting on the evolution of NRPS and PKS gene clusters in Cyanobacteria. Analysis of the phylogenies of C and KS domains from NRPSes and PKSes respectively supported more complex evolution schemes involving gene and domain duplication, indels and/or inversions, gene shuffling, domain recombination as well as domain deletion/substitution [33].

In the second study, genomic data derived from 75 strains of the marine Actinomycete genus *Salinispora* were analyzed for pathways related to polyketide and nonribosomal peptide biosynthesis [34]. The genus *Salinispora* is comprised of only three species, namely *Salinispora pacifica*, *S. arenicola* and *S. tropica*. Like their terrestrial counterparts, marine Actinomycetes are a prolific source of secondary metabolites [35]. Analysis of C and KS domains from the genomes of *Salinispora* strains revealed a high diversity of NRPS and PKS metabolic pathways. Pathways that contained similar gene content and organization were grouped into “operation biosynthetic units” (OBUs). The OBUs were concentrated in genomic islands (GIs) whose boundaries were highly conserved among all strains. These GIs were enriched in mobile genetic elements, suggesting that recent HGT accounts for the majority of identified pathways. Acquisition and transfer events involved, in most cases, complete pathways that subsequently evolved by gene gain, loss and subsequent divergence [34]. Extensive HGT seems to be the main driving force acting upon the evolution of NRPS and PKS pathways in *Salinospora* in contrast to Cyanobacteria. A reasonable explanation for this might be that Cyanobacteria are an ancient lineage of morphologically and metabolically diverse bacteria whereas *Salinospora* is a recently identified bacterial genus comprising only three species. It might be that once new *Salinospora* species and strains are discovered, more complex schemes regarding NRPS and PKS pathway evolution will become apparent.

Further elucidation of the mechanisms driving evolution of NRPS and PKS metabolic pathways could significantly aid the rational human-driven biosynthesis of novel bioactive compounds. Reprogramming of NRPSes and type-I PKSes at the genetic level has been considered to be an attractive alternative to obtain hard-to-synthesize compounds. Initially termed “combinatorial biosynthesis or “pathway engineering”, this approach is now often referred to as “synthetic biology”. The principle and the intention, regardless the definition used, are always similar: by recombination, alteration or exchange of catalytic activities, the encoded reaction cascade is modified to yield novel bioactive compounds or different metabolic profiles [17]. Over the last 15 years several attempts have been reported towards combinatorial biosynthesis of polyketides and nonribosomal peptides. Major obstacles related to limited success of NRPS and PKS reprogramming, such as low availability of metabolic pathway DNA sequences, lack of structural information on catalytic activities and sensitive analytical chemistry technologies, have now been surpassed [17,36]. Recent developments, often mimicking nature itself, in synthetic biology of NRPS and PKS assembly lines include: precursor-directed biosynthesis; engineering of tailoring enzymes; module, domain and subdomain exchanges; active site modifications; and directed evolution of module specificity. These efforts have resulted in hundreds of novel metabolites and it is reasonable to assume that many more are bound to be synthesized in the future [17,37,38,39,40].

## 3. Bioinformatics Tools for the Discovery of Nonribosomal Peptides (NRPs) and Polyketides (PKs)

Recent advances in second and third generations of nucleic acid sequencing technologies like Illumina, Ion proton and Pacific Biosciences (among the most popular) now allow the rapid and low cost whole genome sequencing of bacterial genomes and metagenomes [41,42,43]. This technological leap in combination with ever advancing computational tools for whole genome *de novo* assembly, large-scale gene finding and biochemical pathway predictions have caused a massive generation of microbial genomic data that was unthinkable 20 years ago [41]. At the same time, this flood of data poses a great challenge for the wet-lab and *in silico* bioscientists on how to store, organize and analyze the data and, most importantly, how to distil knowledge. Within this cornucopia of prokaryotic genomes exists a huge and untapped biological body of information regarding secondary metabolites waiting to be exploited.

Genomic and metagenomic data generated by sequencing projects all over the world are freely available and organized in comprehensive updated databases. For example, the NCBI now hosts more than 2,700 complete and annotated prokaryotic genomes that may be freely downloaded and analyzed by anyone. The huge amount of raw sequencing data from genomic and metagenomic projects are stored, organized and are freely accessible in the Sequence Read Archive [44]), also in NCBI. The basic stages in a genomic/metagenomic analysis include *de novo* assembly, gene prediction and functional annotation, with a vast array of available tools that are not the focus of this review. Several institutes apart from NCBI have provided fundamental tools and databases for analyzing microbial genomic/metagenomic data. One of the most prominent is the Joint Genome Institute (JGI), that hosts the Genomes Online database (GOLD), with information about the sequenced genomes [45], whereas the Integrated Microbial Genomes (IMG) computational resources provide the framework for analyzing and reviewing the structural and functional annotations of genomes and metagenomes in a comparative context [46,47]. Other useful metagenomic computational resources include MG-RAST, an open source web application server that performs automatic phylogenetic and functional analysis of metagenomes [48] and CAMERA (J.C. Venter institute) [49].

Once a genome or metagenome has passed the initial stages of assembly and gene prediction, the second phase includes the detection of NRPS and PKS genes and their clusters/pathways [50]. The Basic Local Alignment Search Tool (BLAST) and the profile hidden Markov model suite of tools (HMMER) are widely used algorithms in NRPS and PKS pathway identification. BLAST analysis detects proteins that have homologous regions to known NRPSes or PKSes. Nevertheless, a BLAST-based analysis alone may be misleading, due to the modular nature of NRPS/PKS and the presence of accessory domains that are also found in other unrelated protein families. The optimal approach is based on discovery of conserved domains that are characteristic of NRPSes and PKSes, like the condensation and ketoacyl-synthase domains, respectively. Towards this goal, hidden Markov models of these domains are initially used to detect relevant proteins, with the help of HMMER (as an in-house tool) or the PFAM database/web server [8,50]. Once the candidate targets have been shortlisted, they may be scanned with the whole PFAM database (http://pfam.xfam.org/), in order to identify other domains (basic or accessory) and understand the structure and organization of the modules [51].

Several groups have developed computational tools that broadly focus on detections of secondary metabolite genes and clusters or more specifically focus on detection of NRPS/PKS proteins/pathways. The antiSMASH 3.0 (http://antismash.secondarymetabolites.org/) [52], is a stand-alone tool for the automatic genomic identification and analysis of biosynthetic gene clusters, including NRPS and PKS gene clusters. Its latest version has undergone major improvements. A full integration of the recently developed ClusterFinder algorithm [53] allows the user to employ this probabilistic algorithm to detect putative gene clusters of unknown types. Also, a new de-replication variant of the ClusterBlast module detects similarities of identified clusters to any of 1,172 clusters with known end products. Additionally, active sites of key biosynthetic enzymes are now pinpointed through a curated pattern-matching procedure, whereas chemical structure prediction has been improved by incorporating polyketide reduction states [52]. Given that many biosynthetic pathways span several tens of kb, it is not unusual for such large clusters to be broken in distinct contigs during the assembly process of a draft genome. Therefore, an alternative approach to predict the class of compounds that will be produced is through the use of sequence tags. NaPDoS (http://napdos.ucsd.edu/) [54] was specifically developed using this concept for the classification of PKS and NRPS genes based on sequence tags. Despite small sequence lengths of only 200–300 amino acids, these tags can quite accurately predict pathway types, structural class of the product and in the case of high sequence identity the product structure itself. This approach can be useful in the case of poorly assembled genomes in order to make an initial assessment of the biosynthetic potential of individual strains, including de-replication of well-known compound classes [35,54].

Structural characterization of many catalytic domains in PKSes and NRPSes allowed prediction of their function. For instance, substrate selectivity of the A domains in NRPS is determined by key residues forming the binding cavity which were identified for roughly 50 different substrates thus allowing the building of reliable predictive models. In the case of PKs, substrate redundancy makes reliable prediction somewhat more challenging [50]. Nevertheless, refined predictive models were incorporated in antiSMASH, and NP.searcher [55] as well as in more specific tools like NRPSpredictor2 (nrps.informatik.uni-tuebingen.de) [56], SBSPKS (http://www.nii.ac.in/~pksdb/sbspks/master.html) [57] and NRPS-PKS-substrate-predictor (http://www.cmbi.ru.nl/NRPS-PKS-substrate-predictor/) [58]. Prediction of putative NRPs can be complemented by using a highly informative database such as NORINE (http://bioinfo.lifl.fr/norine/). NORINE is a database specialized in manually curated information on 1,178 NRPs (last visited 26 January 2016). Queries may be issued on compound names, activity, structure, molecular weight, number of amino acid monomers as well as literature references and producing organisms [59,60]. Another useful database of microbial polyketide and nonribosomal peptide gene clusters is the ClusterMine360 (www.clustermine360.ca). It takes advantage of crowd-sourcing by allowing researchers to make contributions into a sequence repository while automation is used to ensure high data consistency and quality. Furthermore, Clustermine360 is a powerful tool for phylogenetic analysis [61]. NORINE and ClusterMine360 significantly facilitate de-replication, thereby aiding the discovery of novel NRPs and PKSes encoded by cryptic or orphan gene clusters.

## 4. Discovery of NRPSes and Type-I PKSes Derived from Marine Microbiomes through Genome Mining

Historically, bacterial natural product discovery relied heavily on luck. High-throughput screening of thousands of strains using a limited number of culture conditions was the main discovery strategy in the hope that a minimum number of strains would yield compounds of interest. Success rate was increased by using improved culture techniques or targeting poorly studied bacterial taxa. However, it became apparent that this strategy was not tenable as the rates of new compound discovery dropped to levels that could not be afforded by the pharmaceutical industry [35]. With the onset of the Genomic Era, an explosion of shotgun sequencing projects occurred. They were amenable to so-called genome mining (GM) that is mining biologically meaningful information based on genomic datasets. GM shifted the paradigm of natural product discovery because now it is feasible to discover natural products from DNA sequences without their prior isolation and structural characterization. Complete or draft genomes of many microorganisms can be analyzed simultaneously by using robust bioinformatics tools, thus leading to identification of novel biosynthetic pathways including those of NRPs and PKs. Prediction of novel biosynthetic pathways and their encoded metabolites harbored in microbial genomes rationalized natural product discovery which is no longer relied upon for “finding the needle in the haystack” [62,63,64,65,66]. Arguably, the most intensively genome-mined marine microbiomes include Cyanobacteria and Actinomycetes such as *Salinispora*. The analysis of their genomes has revealed that even well-studied bacteria can maintain the genetic potential to produce many more secondary metabolites than previously believed [5,33,34]. Distribution of marine Actinomycete-derived 16S rRNA gene sequences has revealed that marine sediment is the main source of these bacteria. Nevertheless, Actinomycetes were also found in association with different marine invertebrates such as soft corals, tunicates and sponges. The microbial biomass can occupy up to 35% of their volume in certain sponge species. It is therefore unsurprising that roughly 22% of 411 natural products from marine Actinomycetes were actually obtained from sponge-associated Actinomycetes. Among the several Actinomycete genera, *Streptomyces*, *Rhodococcus*, *Salinispora* and *Micromonospora* were the most prolific producers of secondary metabolites which displayed broad chemical diversity and interesting bioactivities relevant to medical applications [67,68]. Marine Gram-negative Proteobacteria have generally been thought to possess less metabolic potential for the production of bioactive compounds compared to Cyanobacteria and Actinomycetes; however, several potent antimicrobial NRPs and PKs have been isolated from the marine genus *Pseudoalteromonas* and more recently also from strains of the Roseobacter clade, Vibrionaceae and marine Myxobacteria [6,69,70]. Recently, the genomes of 21 marine α- and γ-Proteobacteria were sequenced and mined for natural product encoding gene clusters. The genome size varied between 4 and 6.2 Mb. Independently of genome size, bacteria of all tested genera carried a significant number of gene clusters encoding secondary metabolites especially within the *Vibrionaceae* and *Pseudoalteromonadaceae* families. A very high potential was identified in pseudoalteromonads with up to 20 gene clusters in a single strain, mostly NRPSes and NRPS-PKS hybrids [71].

In order to quickly assess the metabolic potential harbored in marine prokaryotes for production of NRPs and PKs, within the framework of this review we carried out a rudimentary genome mining of ~2700 complete prokaryotic genomes, downloaded from NCBI. Originally, the PFAM hidden Markov models of condensation (C) and keto-acyl synthase (KS) domains were used to scan all downloaded genomes for NRPSes and PKSes (e-value cutoff: 1e-4) with the HMMER suite of tools in a local PC. Proteins that carried at least one condensation domain were retained as NRPS. Proteins that carried two or more KS domains were further manually inspected, regarding their NCBI gene annotation, to exclude other types of enzymes apart from type-I PKSes that carry KS domains. The list of NRPS and PKS carrying genomes was manually inspected to select known marine prokaryotes. Next, the shortlisted proteins of marine prokaryotes were scanned against the whole PFAM database (in a local PC) with the HMMER software. This time, the cutoff for any domain was stricter (e-value: 1 × 10^−10^; i-value: 1 × 10^−5^). In addition, custom perl scripts were used to remove ambiguity if two or more homologous PFAM domains (usually of the same Clan, *i.e.*, KS and Thiolase domains) were found overlapping in the same protein region. Only the overlapping PFAM domain with the best e-value was retained in such cases. Finally, these filtered proteins and domains were manually inspected again, so as to retain NRPS and type-I PKS genes only. The results of our analysis are summarized in Table 1 (see also supplementary file). In total, 46 marine complete genomes were identified that matched our criteria. Within them, there existed 288 proteins with 563 condensation or/and keto-acyl synthase domains. Interestingly, the vast majority (85%–90%) of domains and proteins were of NRPS (or hybrid NRPS/PKS) origin. The largest megasynthase was an NRPS 9,858 amino acids long, in *Mycobacterium marinum*, with nine modules. The largest PKS was 7279 amino acids long, in *Salinispora arenicola* CNS-205, with five modules. *Nostoc punctiforme*, *Mycobacterium marinum*, and *Salinispora arenicola* were the genomes with the most NRPS and PKS genes, with 29, 24 and 21 genes, respectively. Actually, the extraordinarily high potential of *Mycobacterium marinum* is quite surprising because it is an opportunistic pathogen with greatly underexplored metabolic potential. As expected, the number of NRPS/PKS genes/domains within strains of a species were variable, due to horizontal gene transfer or gene loss, as observed in *Vibrio cholerae*. Furthermore, the gene content was even more dynamic within the genus level, as observed in *Marinomonas*, *Nostoc*, *Oscillatoria*, *Salinispora*, and *Vibrio*.

Current advances in mass spectrometry coupled with genome mining are widely used to discover novel bioactive compounds produced by microorganisms. Molecular networking is a tandem mass spectrometry (MS/MS)-based computational approach that allows for high-throughput multi-strain comparisons thus significantly improving de-replication and identification of new compounds with known structural scaffolds [72,73]. Application of molecular networking to the analysis of 35 closely related *Salinispora* strains including 30 for which genome sequence data were available, was used to recognize previously identified secondary metabolites to generate bioinformatics links between unidentified parent ions and their putative biosynthetic gene clusters and to prioritize compounds for isolation and structure. Furthermore, using genome mining in combination with peptidogenomics, an NRPS gene cluster was linked to a specific parent ion which was targeted for isolation and subsequently identified as retimycin A, a new depsipeptide similar to thicoraline (Table 2) [73]. Thiocoraline is an antitumor compound produced by two marine Actinomycetes taxonomically classified as *Micromonospora* spp. [74].

Recently, several halophilic myxobacterial strains were found to be excellent producers of novel secondary metabolites such as PKs, NRPs and their hybrids [69,75]. Haliamide is a recently characterized metabolite produced by the marine Myxobacterium *Haliangium ochraceum* (Table 2). This new compound was isolated from bacterial cells and its structure was elucidated by mass spectrometry (MS) and nuclear magnetic resonance (NMR). Feeding experiments using labeled putative biosynthetic precursors of haliamide were carried out in order to aid elucidation of its biosynthetic machinery. Mining of *H. ochraceum* genome revealed a biosynthetic gene cluster, attributed to haliamide biosynthesis, which contains one NRPS module followed by four PKS modules. These modules are located on an NRPS/PKS hybrid gene followed by one PKS gene spanning a region of 21.7 kbp. Bioassays were performed in order to evaluate bioactivities of haliamide. Haliamide did not demonstrate any antifungal or antibacterial activity but instead a moderate cytotoxic effect was observed. Interestingly, five more PKS-NRPS gene clusters were identified in the genome of *H. ochraceum* indicating that the metabolic potential of this marine Myxobacterium should be further investigated [76].

Genome mining of the marine Actinomycete *S. tropica* in combination with MS and NMR led to the discovery of salinilactam A (Table 2). Sequence analysis revealed the *slm* gene cluster which is the largest *S. tropica* biosynthetic cluster consisting of six genes that encode a 10-module PKS. Initial bioinformatics analysis of *slm* gene cluster suggested that it coded for a novel polyene macrolactam polyketide. Culture broth of *S. tropica* was inspected for compounds with characteristic UV chromophores associated with polyene units, which led to the isolation of a series of polyene macrolactams exemplified by salinilactam A. Further NMR and MS characterization of salinilactam A revealed a polyene macrolactam framework that was consistent only with the *slm* gene cluster [77]. Recently, a new polyene macrolactam named micromonolactam was isolated from two marine-derived *Micromonospora* species. This new polyene metabolite is a constitutional isomer of salinilactam A but contains a different polyene pattern and one *cis* double bond in contrast to the all *trans* structure reported for salinilactam A. The molecular analysis data also established that micromonolactam is a hybrid polyketide derived from eleven polyketide units and a modified amino acid unit [78].

In another study, salinosporamide K (Table 2), a potent antitumor compound, was discovered by comparative genomics of *S. pacifica* genome with that of *S. tropica*. An incomplete biosynthetic gene cluster was identified in the draft genome of *S. pacifica* which was orthologous to the 41 kb salinosporamide A gene cluster in *S. tropica*. Initially, reverse-transcriptase PCR of selected genes located on the putative salinosporamide K cluster was performed in order to confirm its transcription. Further isolation and analysis by NMR confirmed the structure of salinosporamide K [79].

Identification of NRPS and PKS gene clusters in genomes of culturable microorganisms is a crucial step in natural product discovery but, as a matter of fact, most biosynthetic gene clusters remain silent when microorganisms are grown in the laboratory. Releasing this silent potential represents a significant technological challenge [80,81]. Although our understanding of the physiological and nutrient factors which pervade in marine ecosystems is partial at best, recent advances regarding activation of silent biosynthetic gene clusters are promising [80]. Employment of the “OSMAC” approach, that is the ability of single strains to produce different secondary metabolites when growing under distinct culture conditions, has been shown to alter secondary metabolism in marine bacteria and fungi [82,83]. Furthermore, co-cultivation of marine microorganism activates silent biosynthetic clusters either through competition or synergistic interplay [80]. In addition to the cultivation parameters, external physical or chemical cues induce the expression of silent biosynthetic clusters or increase their product yields in microorganisms. The discovery of quorum sensing clearly has demonstrated that marine microorganisms form complex communities where cell-to-cell communication influences physiological and metabolic responses in diverse environments [80,84,85]. Therefore, application of signaling molecules, such as lactones or antibiotics at sub-inhibitory concentrations, might elicit activation of silent gene clusters. While the identification of biosynthetic gene clusters, through genome mining, has already expanded our knowledge regarding the biosynthetic ability of microorganisms, the development of multiple approaches to elicit the production of unknown secondary metabolites remains a key challenge in bioprospecting of cryptic biosynthetic pathways [80].

## 5. Discovery of NRPSes and Type-I PKSes Derived from Marine Microbiomes through Metagenomics

**Figure 2:**
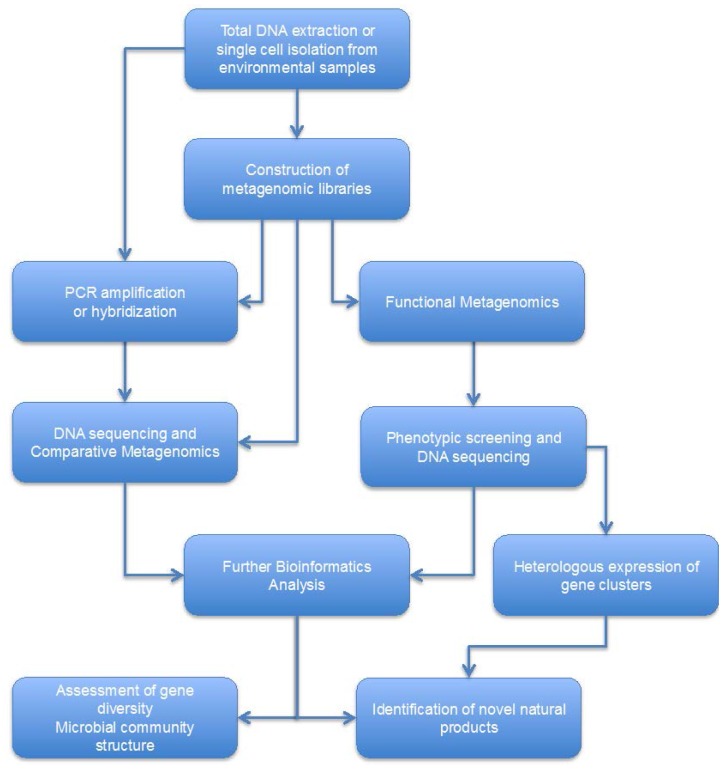
General workflow in metagenomics. Sequenced-based and/or functional approaches are commonly adopted in order to discover new natural products, to study microbial communities or to sequence whole genomes of unculturable single cells.

Although a plethora of natural products have been isolated from cultured marine microorganisms [1,4,5,6], most of them (especially symbionts) remain unculturable. It is well established that only a very small fraction of microbial biodiversity, found in any given niche, can be cultured in the laboratory [91,92,93]. The simplest explanation for why most bacteria are not growing in the laboratory is that microbiologists are failing to mimic essential aspects of their environment. Attempting to simultaneously replicate environmental aspects such as nutrients, pH, osmotic conditions and temperature, results in myriads of possibilities that cannot be exhaustively addressed with reasonable time and effort [91,92]. On the other hand, discovery of natural products could definitely be accelerated if unculturable microorganisms become culturable [94]. A recent example supporting this view is the discovery of teixobactin, a novel antibiotic that inhibits cell wall synthesis by binding to highly conserved motifs of peptidoglycan and teichoic acid precursors [95]. Different approaches to culturing the missing bacterial diversity have been described [3,92]. Simulating the environment seems to be the most promising. Several research groups designed diffusion chambers where bacteria of interest could be cultivated *in situ*. These bacteria were separated from the general microbial population but still nutrients, growth factors and environmental cues were available. Microscopic examination of the chambers revealed microcolonies of bacteria growing within them, the majority of which could be further isolated and propagated by re-inoculation into fresh chambers [92,96,97,98]. Ongoing progress in this field will definitely expand our technical capacity to culture microbes in the laboratory, even though most of them will remain unculturable, at least in the near future. Metagenomics is a rapidly growing research field used to study directly microbial (meta) genomes and their products. The distinct advantage of metagenomics is that it allows the study of unculturable or yet-uncultured microbes which are part of microbial communities present in environmental samples (e.g., soil, sea water) or hosts (e.g., plants, insects, human) [2,99,100,101,102]. Furthermore, single cell genomics (SCG) is unique among current genomic approaches in yielding access to the genomes of individual cells isolated directly from environmental samples, without the complications of culture or compositing data from multiple cells or strains. Flow cytometry and fluorescence-activated cell sorting (FACS) are widely used in SCG projects, for single cell isolation from diverse environments. Subsequently, whole genome amplification of isolated cells (via multiple displacement amplification) is commonly achieved and NGS technologies combined with bioinformatics tools are used to analyze and assemble the genomes. [103]. The general workflow in metagenomic projects is shown in Figure 2. Total DNA extraction or single cell isolation is the first step. Technical issues such as biased sampling, contamination or low DNA yield can jeopardize metagenomic projects. Extracted DNA is used to construct metagenomic libraries in appropriate vectors (e.g., cosmids, fosmids, BACs) or it can be used as template in PCR amplification of specific gene groups (e.g., 16S rRNA gene, C or KS domains of NRPSes and KSes). PCR amplicons can be directly sequenced using NGS technologies and the DNA sequences can be analyzed by comparative metagenomics. Comparative metagenomics are often used in order to assess gene diversity, to identify biosynthetic gene clusters in certain environments or to elucidate the structure of microbial communities. This approach has been applied in the majority of metagenomic projects because heterologous gene expression in host strains is not required. On the other hand, in functional metagenomics, constructed libraries are screened in order to identify specific phenotypes such as antibiotic or enzyme production. As soon as the appropriate function is identified, the clone which contains the gene(s) is sequenced and DNA sequences are further analyzed. In this way, new gene classes of known or even unknown functions can be identified [104,105,106]. In most cases heterologous expression of biosynthetic gene clusters is attempted. The *E. coli* has been the host of choice; however, different bacterial strains from *Streptomyces*, *Pseudomonas* and *Bacillus* genera have been employed as hosts [107]. Heterologous expression could be hampered due to a variety of factors such as large size of biosynthetic clusters, differences in codon usage, lack of regulating elements and abnormalities in protein folding [2,104,107]. Moreover, toxicity of the gene product as well as impaired protein secretion can be major drawbacks of heterologous gene expression [104,107].

Marine invertebrates such as sponges, soft corals and tunicates are known to contain (as *epi*- or endosymbionts) large amounts of phylogenetically diverse microorganisms. These microbiomes are currently underexplored regarding their metabolic potential although it is established that their hosts are prolific producers of natural products [2,4,108,109]. Metagenomic approaches are widely adopted in order to assess NRPS and PKS gene diversity of such microbiomes [86,88,110,111,112]. These studies revealed previously unknown gene classes. For instance, Della Sala *et al.*, using DNA extracted from the microbiome of a sponge, amplified fragments of PKSes by PCR and then pyrosequenced these fragments [88]. Sequence analysis has demonstrated that dominant PKS genes belong to *supA* and *swfA* groups. Moreover, several non-*supA* and non-*swfA* type-I PKS fragments were identified. A significant portion of these fragments resembled type-I PKSes from protists, suggesting, for the first time, that bacteria may not be the only source of polyketides from sponges [88].

Similarly, Woodhouse *et al.* assessed the diversity of NRPS and PKS genes within the microbiomes of six Australian marine sponge species, by whole-genome shotgun pyrosequencing [111]. In this way, 100 novel KS domain sequences and 400 novel C domain sequences were identified [111].

Furthermore, metagenomic approaches have been successfully employed for discovery and isolation of novel bioactive compounds synthesized by NRPSes and PKSes derived from marine microbiomes. Bryostatin 1 is a polyketide (Table 2) which belongs to the family of macrocyclic lactones. It was initially detected in extracts from the marine bryozoan *Bugula neritina* and has been extensively tested for its antitumor activity and as an anti-Alzheimer’s drug [2,87]. Later it was demonstrated that bryostatin is actually produced by a particular symbiont in the larvae of the bryozoan which was identified and proposed to be a novel γ-Proteobacterium, “*Candidatus* Endobugula sertula” [90]. In 2004, Hildebrand *et al.* identified the bryostatin PKS gene cluster in “*Candidatus* Endobugula sertula” [113]. Total DNA was the template for PCR amplification, using degenerated primers, thus leading to amplification of 300 bp products of the β-ketoacyl synthase (KSa). These amplicons were used as probes in hybridization of metagenomic library clones. The 65 kb gene cluster of bryostatin was identified and genetic analysis of the *bryA* revealed that it is responsible for the initial steps of bryostatin biosynthesis [113]. Interestingly, *trans*-AT PKSes are involved in biosynthesis of PKs derived from marine microbiomes, particularly those with biosynthesis attributed to endosymbionts of marine invertebrates such as sponges [18,32]. Ecteinascidin 743 (ET-743) is now an approved antitumor agent that has been isolated from the Caribbean mangrove tunicate *Ecteinascidia turbinata*. [1,2]. Structural similarity to three other bacterial-derived natural products (safamycins) suggested that ET-743 was actually produced by a bacterial symbiont. Using metagenomic sequencing of total DNA from the microbiome associated with the tunicate resulted in the identification of the biosynthetic gene cluster which encodes ET-743. This gene cluster spans 35 kb and contains 25 genes that comprise the core of an NRPS biosynthetic pathway. Rigorous sequence analysis based on codon usage of two large unlinked contigs suggests that “*Candidatus* Endoecteinascidia frumentensis” produces the ET-743 metabolite [114]. Recently, the whole genome of this new bacterium was sequenced and assembled directly from metagenomic DNA isolated from the tunicate. Analysis of the genome revealed strong evidence of an endosymbiotic lifestyle and extreme genome reduction. Phylogenetic analysis suggested that this bacterium is taxonomically distinct from other sequenced bacteria and it could represent a new family of γ-Proteobacteria [89].

Pederin is an antitumor agent produced by an uncultured bacterial symbiont of *Paederus fuscipes* beetles. The complete NRPS-PKS biosynthetic cluster of pederin was identified though sequence-based analysis and metagenomic library screening. Later on a pederin-informed survey of PCR amplicons from the Japanese sponge *Theonella swinhoei* metagenome identified three PKSes which corresponded to biosynthetic pathways of the potent antitumor agents onnamides (Table 2) [7,115,116].

Apratoxins are potent antitumor agents produced by Cyanobacteria [117,118,119]. A dual-method approach of single-cell genomic sequencing based on multiple displacement amplification and metagenomic library screening has been employed in order to identify the apratoxin A biosynthetic gene cluster. The roughly 58 kb biosynthetic gene cluster is composed of 12 open reading frames and has a type I modular mixed polyketide synthase/nonribosomal peptide synthetase (PKS/NRPS) organization and features loading and off-loading domain architecture never previously described [119].

## 6. Concluding Remarks

There is an increasing need for discovery of novel or modified bioactive compounds with applications in medicine, human wellbeing, sustainable development and environmental protection. Bioprospecting of the enormous biodiversity hidden in marine ecosystems opens new avenues. Currently, attention is turning to marine microbiomes as an untapped source of novel natural products, such as nonribosomal peptides and polyketides. These are secondary metabolites synthesized by microorganisms that enhance their adaptation in diverse environments. The biosynthetic machinery of these metabolites consists of megasynthases, namely NRPSes and PKSes. The advent of the Genomic and Metagenomic Era has revolutionized natural product discovery strategies. NGS technologies dropped dramatically the cost of sequencing and at the same time increased exponentially our technical capacity to obtain genomic datasets. With the wealth of minable genomic data now available to scientists, bioinformatics is essential. The last few years, robust and sophisticated computational tools have been developed to identify NRPS and PKS pathways though genome mining. Nevertheless, *in silico* prediction of NRP and PK structures needs further improvements. New findings regarding the mechanisms underlying NRPS and PKS evolution, illustrate how stochastic events such as gene duplication or domain deletion/shuffling allows microorganisms to rapidly expand and adapt their metabolic potential. It is conceivable that mimicking nature’s evolutionary mechanisms in the laboratory will accelerate discovery of either novel or modified NRPs and PKs. Despite metagenomics being a relatively new research tool, its pivotal role is already well appreciated in natural product discovery from unculturable or yet-uncultured marine microbiomes. Recent advances in metabolomics (molecular networking, peptidogenomics) complement metagenomics consequently making targeted metagenomics of NRPs and PKs now a reality. In order to maximize our ability to harvest marine resources, interdisciplinary approaches should be adopted. Further developments in (meta)genomics, bioinformatics, biosynthetic pathway reprogramming, metabolomics and natural product chemistry will surely fuel the discovery pipeline of NRPs and PKs from the marine microbiomes in the foreseeable future.

## Figures and Tables

**Table 1:** NRPS and PKS content in 46 marine prokaryotic genomes after applying a set of filters. Of note, the number of C and KS domains corresponds only to filtered proteins.

Species	C and KS Domains	C Domains	Total NRPS and PKS Proteins	NRPS (or NRPS/PKS Hybrid) Proteins
*Acaryochloris marina* MBIC11017	10	10	6	6
*Alteromonas macleodii* AltDE1	16	16	10	10
*Alteromonas macleodii* str. “Ionian Sea U4”	16	16	10	10
*Alteromonas macleodii* str. “Ionian Sea U7”	16	16	10	10
*Alteromonas macleodii* str. “Ionian Sea U8”	16	16	10	10
*Alteromonas macleodii* str. “Ionian Sea UM7”	16	16	10	10
*Haliangium ochraceum* DSM 14365	41	24	15	9
*Halomonas elongata* DSM 2581	4	4	2	2
*Marinomonas mediterranea* MMB-1	14	14	4	4
*Marinomonas posidonica* IVIA-Po-181	3	3	1	1
*Marinomonas* sp. MWYL1	18	18	8	8
*Mycobacterium marinum* M	73	64	24	21
*Nostoc azollae* 0708	3	3	1	1
*Nostoc punctiforme* PCC 73102	61	52	29	25
*Nostoc* sp. PCC 7107	9	7	7	7
*Nostoc* sp. PCC 7120	18	17	10	10
*Nostoc* sp. PCC 7524	3	1	2	1
*Oscillatoria acuminata* PCC 6304	14	14	10	10
*Oscillatoria nigro-viridis* PCC 7112	16	16	12	12
*Phaeobacter gallaeciensis* 2.10	1	1	1	1
*Phaeobacter gallaeciensis* DSM 26640	1	1	1	1
*Phaeobacter inhibens* DSM 17395	1	1	1	1
*Photobacterium profundum* SS9	9	8	4	4
*Planctomyces brasiliensis* DSM 5305	2	0	1	0
*Planctomyces limnophilus* DSM 3776	2	0	1	0
*Prochlorococcus marinus* str. MIT 9303	1	1	1	1
*Pseudovibrio* sp. FO-BEG1	9	7	5	5
*Salinispora arenicola* CNS-205	42	25	21	16
*Salinispora tropica* CNB-440	28	16	19	15
*Synechococcus* sp. PCC 7502	2	2	1	1
*Vibrio alginolyticus* NBRC 15630 = ATCC 17749	3	3	1	1
*Vibrio anguillarum* 775	3	3	2	2
*Vibrio campbellii* ATCC BAA-1116	9	9	7	7
*Vibrio cholerae* IEC224	5	5	2	2
*Vibrio cholerae* LMA3984-4	5	5	2	2
*Vibrio cholerae* M66-2	5	5	2	2
*Vibrio cholerae* MJ-1236	5	5	2	2
*Vibrio cholerae* O1 biovar El Tor str. N16961	5	5	2	2
*Vibrio cholerae* O1 str. 2010EL-1786	5	5	2	2
*Vibrio cholerae* O395	10	10	4	4
*Vibrio furnissii* NCTC 11218	5	5	3	3
*Vibrio nigripulchritudo*	25	24	12	12
*Vibrio* sp. EJY3	1	1	1	1
*Vibrio vulnificus* CMCP6	4	4	3	3
*Vibrio vulnificus* MO6-24/O	4	4	3	3
*Vibrio vulnificus* YJ016	4	4	3	3
Total	563	486	288	263

**Table 2:** NRPs and PKs compounds derived from marine microbiomes discovered though genome mining and metagenomics.

Compound	Enzyme	Discovering Approach	Microbial Source	Mode of Action	Reference
Haliamide	PKS-NRPS	Genome mining	*Haliangium ochraceum*	Cytotoxic	[71]
Salinosporamide K	NRPS	Genome mining	*Salinispora pacifica*	Antitumor	[74]
Retimycin A	NRPS	Genome mining	*Sallinispora arenicola*	Antitumor	[68]
Salinilactam A	PKS	Genome mining	*Salinispora tropica*	Antibiotic	[72]
ET-743	NRPS	Metagenomics	*“Candidatus Endoecteinascidia frumentensis”*	Antitumor	[86]
Pederin	PKS	Metagenomics	*Paederus fuscipes* metagenome	Antitumor	[87]
Bryostatin	PKS	Metagenomics	*Candidatus* Endobugula sertula	Antitumor	[88]
Apratoxin A	PKS-NRPS	Metagenomics	*Lyngbya bouillonii*	Antitumor	[89]
Onnamide	PKS-NRPS	Metagenomics	*Theonella* *swinhoei* metagenome	Antitumor	[90]

## Supplementary Files

Supplementary File 1
